# Impact of COVID-19 pandemic on HbA_1c_ management and results in pediatric and adult outpatients with diabetes

**DOI:** 10.1515/almed-2022-0098

**Published:** 2023-02-28

**Authors:** Paloma Oliver, Marina Pellicer, Daniel Prieto, Jorge Diaz-Garzon, Roberto Mora, Ileana Tomoiu, Noemi Gonzalez, Atilano Carcavilla, Isabel Gonzalez-Casado, Itsaso Losantos, Antonio Buño, Pilar Fernandez-Calle

**Affiliations:** Department of Laboratory Medicine, La Paz University Hospital, Madrid, Spain; Department of Endocrinology, La Paz University Hospital, Madrid, Spain; Department of Pediatric Endocrinology, La Paz University Hospital, Madrid, Spain; Department of Biostatistics, La Paz University Hospital, Madrid, Spain

**Keywords:** COVID-19, diabetes mellitus, diagnostic techniques and procedures, telemedicine

## Abstract

**Objectives:**

Diabetes mellitus intensify the risks and complications related to COVID-19 infection. A major effect of the pandemic has been a drastic reduction of in-person visits. The aim of this study was to evaluate the impact of the COVID-19 pandemic on HbA_1c_ management and results among pediatric and adult outpatients with diabetes, considering the laboratory and point-of-care testing (POCT) HbA_1c_ measurements.

**Methods:**

Observational retrospective study including patients from pediatric and adult diabetes units was conducted. HbA_1c_ results obtained in the laboratory and POCT over 3 years (2019–2021) were collected from the laboratory information system.

**Results:**

After the lockdown, the number of HbA_1c_ plummeted. Children returned soon to routine clinical practice. The number of HbA_1c_ increased gradually in adults, especially in POCT. Globally, HbA_1c_ results were lower in children compared with adults (p<0.001). HbA_1c_ values in children (p<0.001) and adults (p=0.002) decreased between pre-pandemic and post-pandemic periods, though lower than the HbA_1c_ reference change value. The percentage of HbA_1c_ results above 8% remained stable during the study period.

**Conclusions:**

Continuous glucose monitoring and a telemedicine have been crucial, even allowing for improvements in HbA_1c_ results. During the lockdown, patients with better metabolic control were managed in the laboratory whereas patients with poorer control or a severe clinical situation were attended in diabetes units by POCT. Adults returned to pre-pandemic management slowly because they were more susceptible to morbidity and mortality due to COVID-19. Coordination among all health professionals has been essential to offering the best management, especially in difficult scenarios such as the COVID-19 pandemic.

## Introduction

Diabetes mellitus (DM) is a major cause of morbidity and mortality worldwide. This disease is linked with numerous complications that affect disease severity. There is evidence that high blood glucose and DM play a vital role in the death and infection severity of individuals with infectious viruses, such as the Middle East respiratory syndrome-related coronavirus, H1N1 (influenza A) and severe acute respiratory syndrome-coronavirus. Both type 1 and type 2 DM intensify the risks and complications related to COVID-19 infection, and the risk is even higher in those of older age [1].

One of the effects of the COVID-19 pandemic on the healthcare system has been a drastic reduction of in-person visits, raising concerns about how this reduction will affect the management and control of chronic patients. A decrease in metabolic control has been found, especially during the lockdown period. In this context, attendance was redirected toward telemedicine to compensate for the declining in-person visits [2]. The reduction of routine visits can result in poorer metabolic control and complications that are not treated in a timely manner [3]. To avoid such complications, it is important that people with diabetes are able to regularly monitor blood glucose levels, attend medical appointments, access the right kind of medicine, be physically active, and follow a healthy diet [4].

Additionally, the average cost of secondary care for people with DM and COVID-19 is higher compared with people with COVID-19 without DM [3]. It also highlights the importance of a greater focus on prevention and adequate treatment of DM and the need for special attention to prevent infection with COVID-19 patients with DM [3].

La Paz University Hospital is a tertiary public hospital located in Madrid that is a referral center, whose specialties are widely recognized for its range of specializations. It is one of the largest hospitals in Spain, with approximately 1,300 beds, with several satellite facilities providing specialist services and 23 primary healthcare centers. The laboratory medicine department of this hospital includes a specific point-of-care testing (POCT) unit that has led the hospital’s POCT network for the last 23 years. This POCT network currently includes 2 HbA_1c_ devices in pediatric and adult diabetes units [5]. The follow up patients with diabetes are managed with HbA_1c_ measurements performed both in the laboratory and in the POCT in our hospital. Therefore, an integrated assessment of HbA_1c_ management and results in both locations is a useful tool for measuring clinical practice being also necessary for an overview of diabetes care.

Since the lockdown that started on March 11, 2020, in Madrid, Spain, routine hospital visits for patients with diabetes had been significantly reduced; thus, the COVID-19 pandemic modified the management of these patients, leading to the implementation of approaches that entailed telemedicine.

The aim of this study was to evaluate the impact of the COVID-19 pandemic on HbA_1c_ management and results in pediatric and adult outpatients with diabetes, considering the laboratory and POCT HbA_1c_ measurements requested.

## Materials and methods

This was an observational retrospective study that included patients from the pediatric and adult diabetes units.

The HbA_1c_ results obtained in the laboratory (Tosoh G11; Horiba Medical) and from POCT (DCA Vantage; Siemens Healthineers) from 2019 to 2021 were collected from the laboratory information system (LabTrak; Intersystems) in an anonymized format.

All these measurements are accredited under the International Organization for Standardization (ISO) 15189 and ISO 22870 standards by the Spanish National Accreditation Body (ENAC). HbA_1c_ results of the DCA Vantage in POCT and the central laboratory were assessed showing interchangeability. Furthermore, internal and external quality assurance have been assessed both in laboratory and POCT to verify that they fulfill the analytical performance specifications established by laboratory.

In addition, the laboratory established suitable key performance indicators to monitor and evaluate the performance of critical aspects of the analytical and extra-analytical processes on a monthly basis.

The variables selected related to HbA_1c_ management and results were as follows:

### HbA_1c_ measurement management

– Total number of HbA_1c_ measurements performed (n).
– Ratio of HbA_1c_ measurements in the laboratory/number of HbA_1c_ POCT measurements (%).
– Total number of patients managed in doctors’ offices (n).
– Ratio of HbA_1c_ measurements/patient (n).

### HbA_1c_ results

– HbA_1c_ results, expressed as mean and SD.
– HbA_1c_ results >8% (expressed as the % of HbA_1c_ results >8% over the total HbA_1c_ results).

### Statistical methods

We considered 2 periods of time for this study: The pre-pandemic period from January 2019 to February 2020 and the post-pandemic period from March 2020 to December 2021. The HbA_1c_ results’ distribution normality was assessed by means of the Kolmogorov–Smirnov test. A descriptive analysis of the HbA_1c_ results was performed with mean and standard deviation. The comparisons of HbA_1c_ results between pediatric and adult patients, as well as between the laboratory and POCT, were performed using Student’s t-test, also taking into consideration the reference change value (RCV) for HbA1c. The RCV was calculated following the formula for classical RCV=2^1/2^ × Z × (CVI^2^ + CVA^2^)^1/2^, with an HbA_1c_ within-subject biological variation (CVI) of 1.6% and a laboratory annual analytical coefficient variation (CVA) of 2.8% for a given probability of 95% [6]. Generalized linear models were used to evaluate the effect of the pandemic on pediatric and adult patients. A p-value less than 0.05 was established as significant.

## Results

### HbA_1c_ measurements management

**Figure 1:**
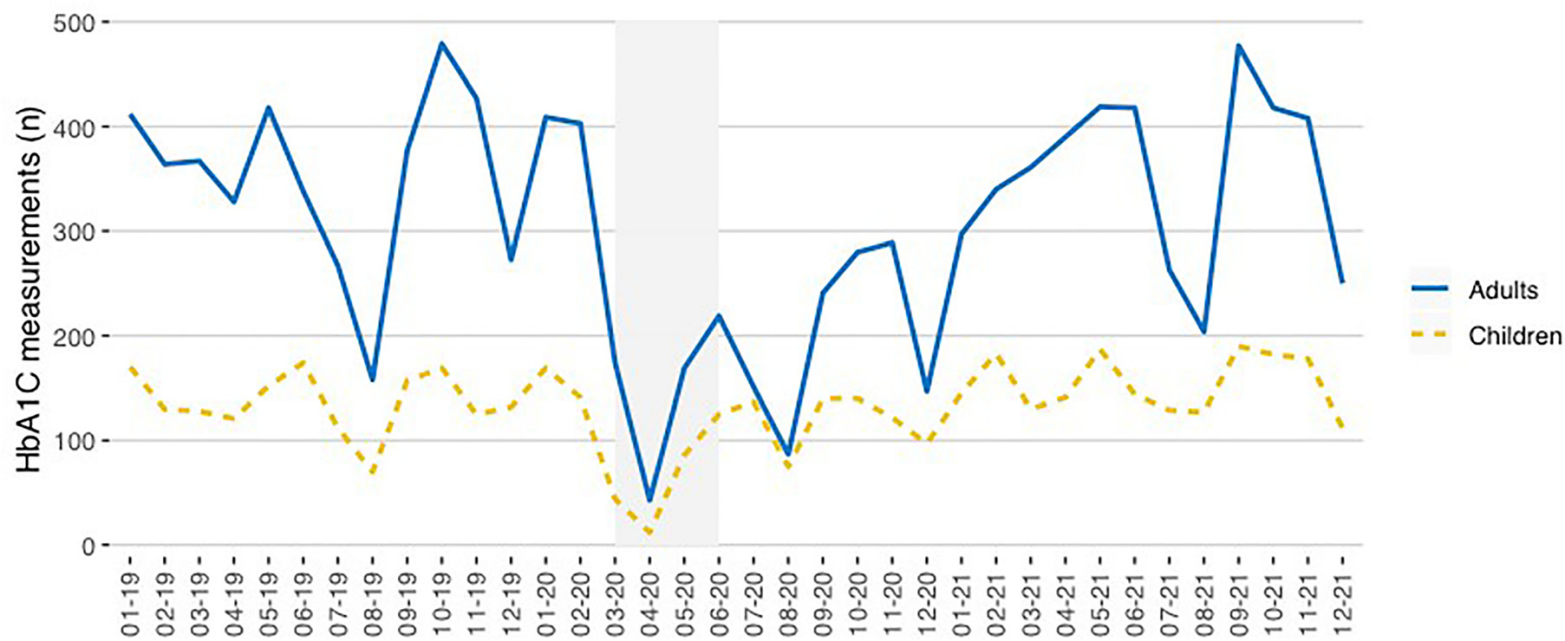
Y axis: Total number of HbA_1c_ measurements (laboratory + POCT) performed in each clinical setting. X axis: Time (month–year). The period of strict lockdown in Madrid corresponds to the shaded area (March–May 2020).

**Figure 2:**
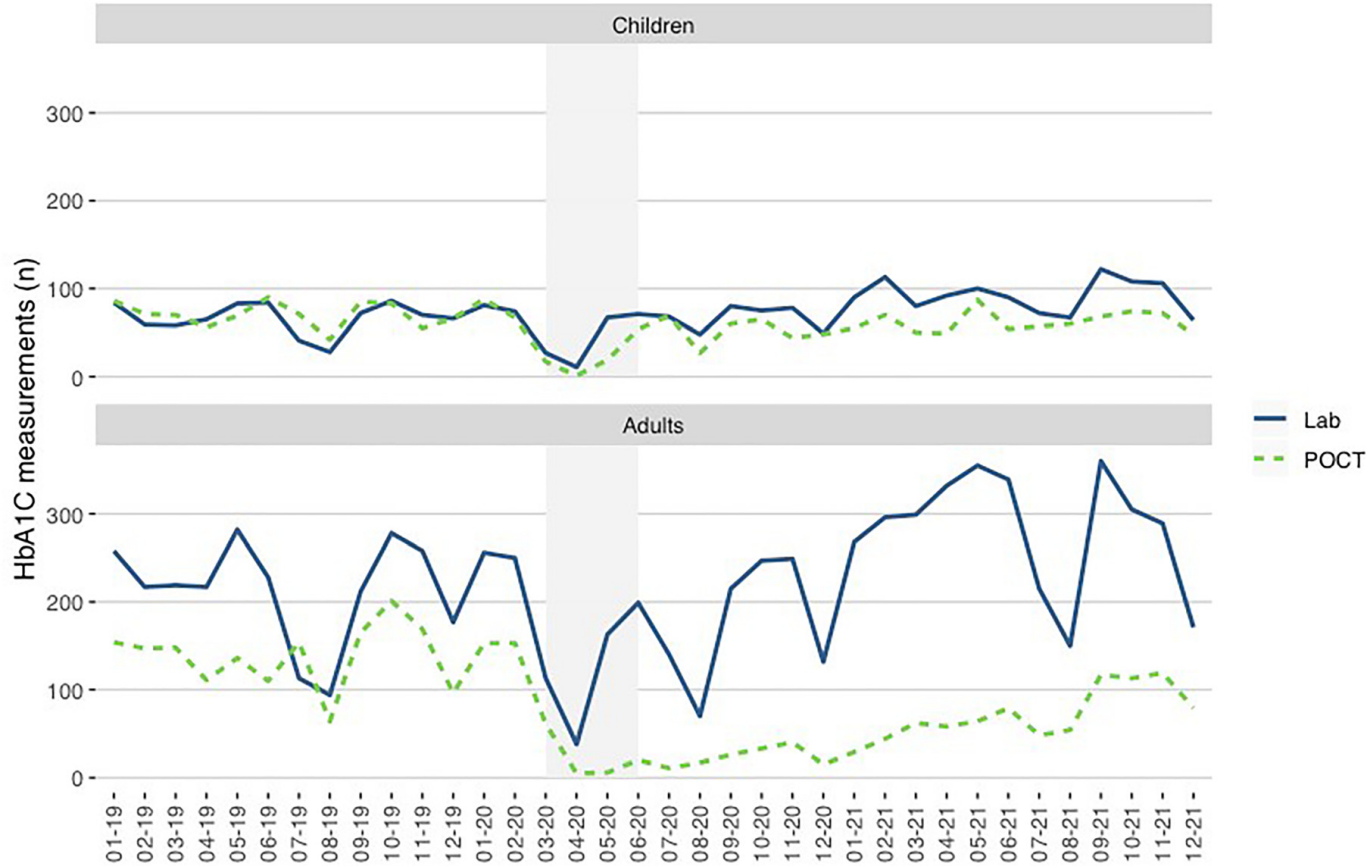
Y axis: Number of HbA_1c_ measurements of pediatric and adult patients in the laboratory and POCT. X axis: Time (month–year). The period of strict lockdown in Madrid corresponds to the shaded area (March–May 2020).

Figures 1 and 2 and Supplementary 1 show the number of HbA_1c_ measurements (laboratory and POCT) performed in the pediatric and adult diabetes units.

A total of 4,776 and 11,065 total HbA_1c_ measurements were performed in the pediatric and adult diabetes units, respectively. A total of 1,042 children and 1,767 adults were attended during the study period, with 1.5 annual measurements per patient in children and 2.1 in adults.

During the strict lockdown (March + April + May), a total of 81 measurements of children were performed in the laboratory (3 + 11 + 67) and 21 in the POCT (1 + 1 + 19). Concerning adults, 221 measurements were performed in the laboratory (20 + 38 + 163) and 11 in the POCT (0 + 5 + 6).

The number of HbA_1c_ measurements was stable in both clinical settings before March 2020. After March 11, 2020, the number of HbA_1c_ measurements plummeted. Figures 1 and 2 and Supplementary 1 show that pediatric patients returned to routine clinical practice in June 2020. However, the number of HbA_1c_ measurements increased more gradually and slowly in adult patients, especially in POCT.

### HbA_1c_ results

**Figure 3:**
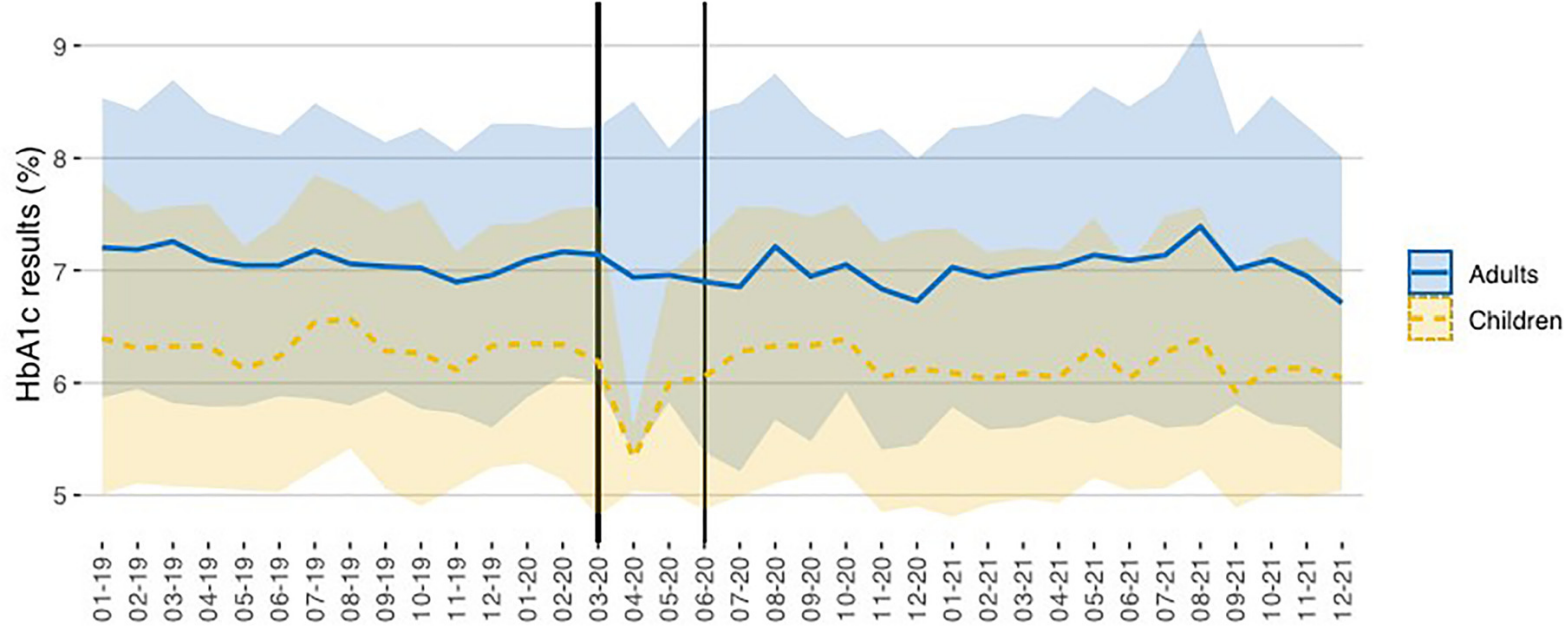
Y axis: The HbA_1c_ results (mean ± 1 SD) obtained in pediatric and adult patients (laboratory + POCT). X axis: Time (month–year). The period of strict lockdown in Madrid corresponds to the shaded area (March–May 2020).

**Figure 4:**
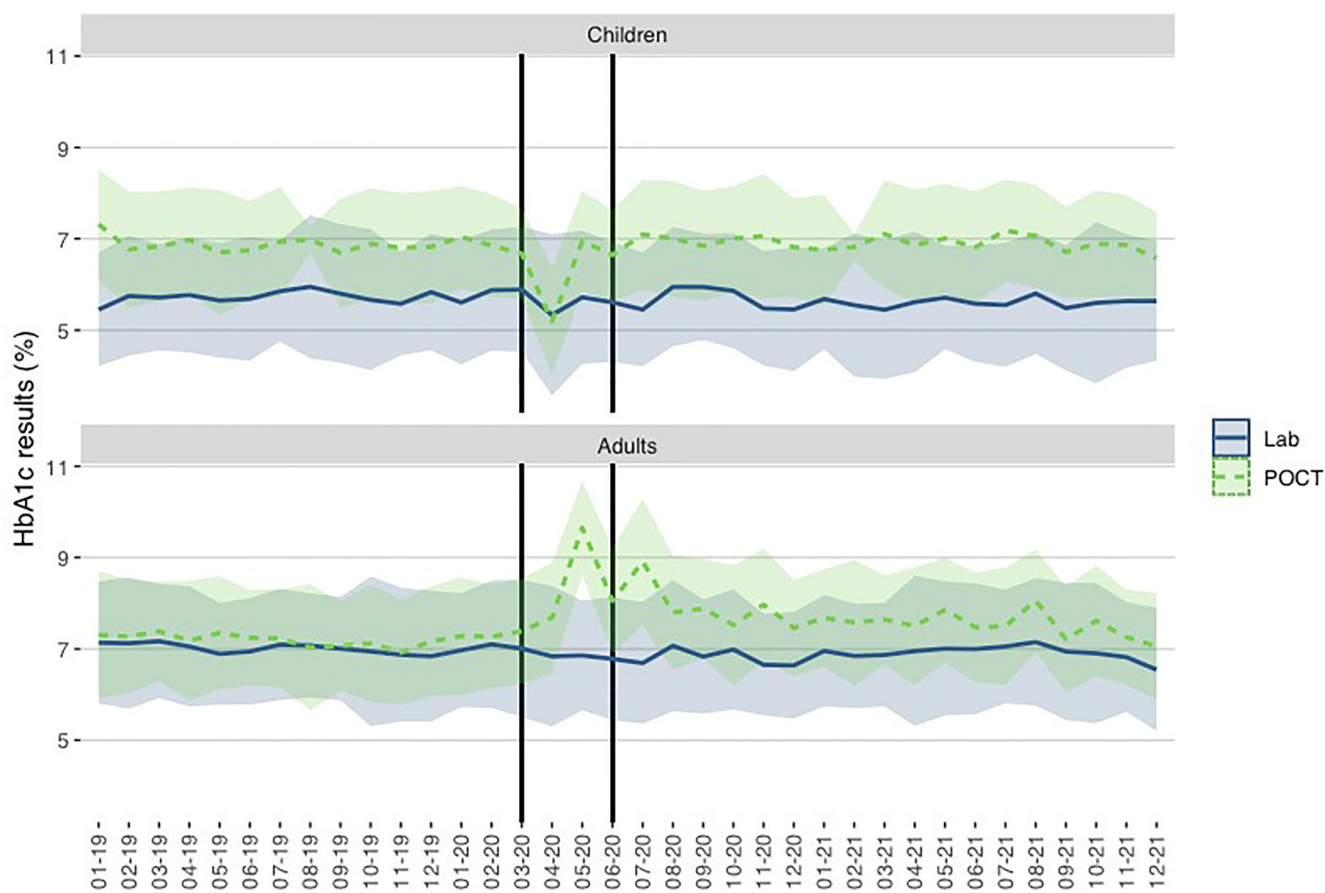
Y axis: HbA_1c_ results (mean ± 1 SD) obtained in pediatric and adult patients in the laboratory and POCT. X axis: Time (month–year). The period of strict lockdown in Madrid corresponds to the shaded area (March–May 2020).

Figures 3 and 4 show the mean concentration and standard deviation (SD) of all HbA_1c_ results (laboratory and POCT) obtained in the pediatric and adult diabetes units during the study period.

In general, the HbA_1c_ results were lower in pediatric patients (6.21%; DS: 1.18) compared with adults (7.05%; DS: 1.32). The difference was significant according to Student’s t-test (p<0.001) and considering 8.9% as the RCV for HbA1c.

Taking into account the pre-pandemic and post-pandemic periods, we observed a significant difference between the HbA_1c_ results in pediatric patients: 6.31% (SD: 1.21) vs. 6.15% (DS: 1.15), p<0.001; and in adult patients: 7.09% (SD: 1.25) vs. 7.01% (SD: 1.39), p=0.002. However, the differences were lower than the RCV.

Considering February 2020 as the reference month in the pre-pandemic period, we observed a significant decrease in the average HbA_1c_ in April (p=0.003), May (p=0.026), and June (p=0.043) in children. From July 2020 on, the results were similar to the pre-pandemic period. For adult patients, we also observed a similar trend in June (p=0.020) and July (p=0.017), returning to the pre-pandemic period from August 2020 on.

When comparing HbA_1c_ values between the laboratory and POCT in children, we observed significant differences in HbA_1c_ results obtained in the laboratory (p=0.001) but not in the POCT (p=0.176) throughout the study period. Taking into account February 2020 as a reference, we did not observe differences in the following months either in the laboratory or in the POCT.

In adults, we observed significant differences in HbA_1c_ results obtained in the laboratory (p=0.001) and in the POCT (p=0.009) throughout the study period. Taking into account February 2020 as a reference, we observed differences in laboratory results in May (p=0.041), June (p=0.005), and July (p=0.001). We also observed differences in POCT in May (p=0.002) and July (p=0.004). From August 2020 on, results returned to the pre-pandemic period.

**Figure 5:**
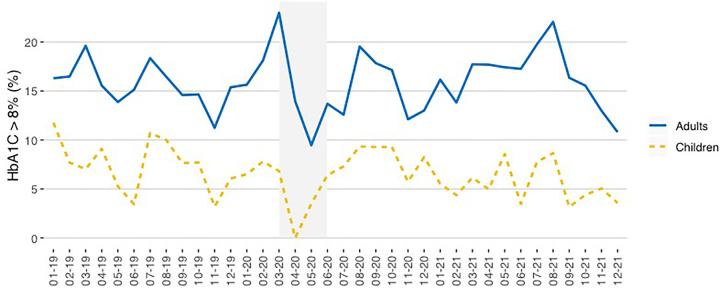
Y axis: Percentage of HbA_1c_ >8% (laboratory and POCT) obtained in pediatric and adult patients. X axis: Time (month–year). The period of strict lockdown in Madrid corresponds to the shaded area (March–May 2020).

**Figure 6:**
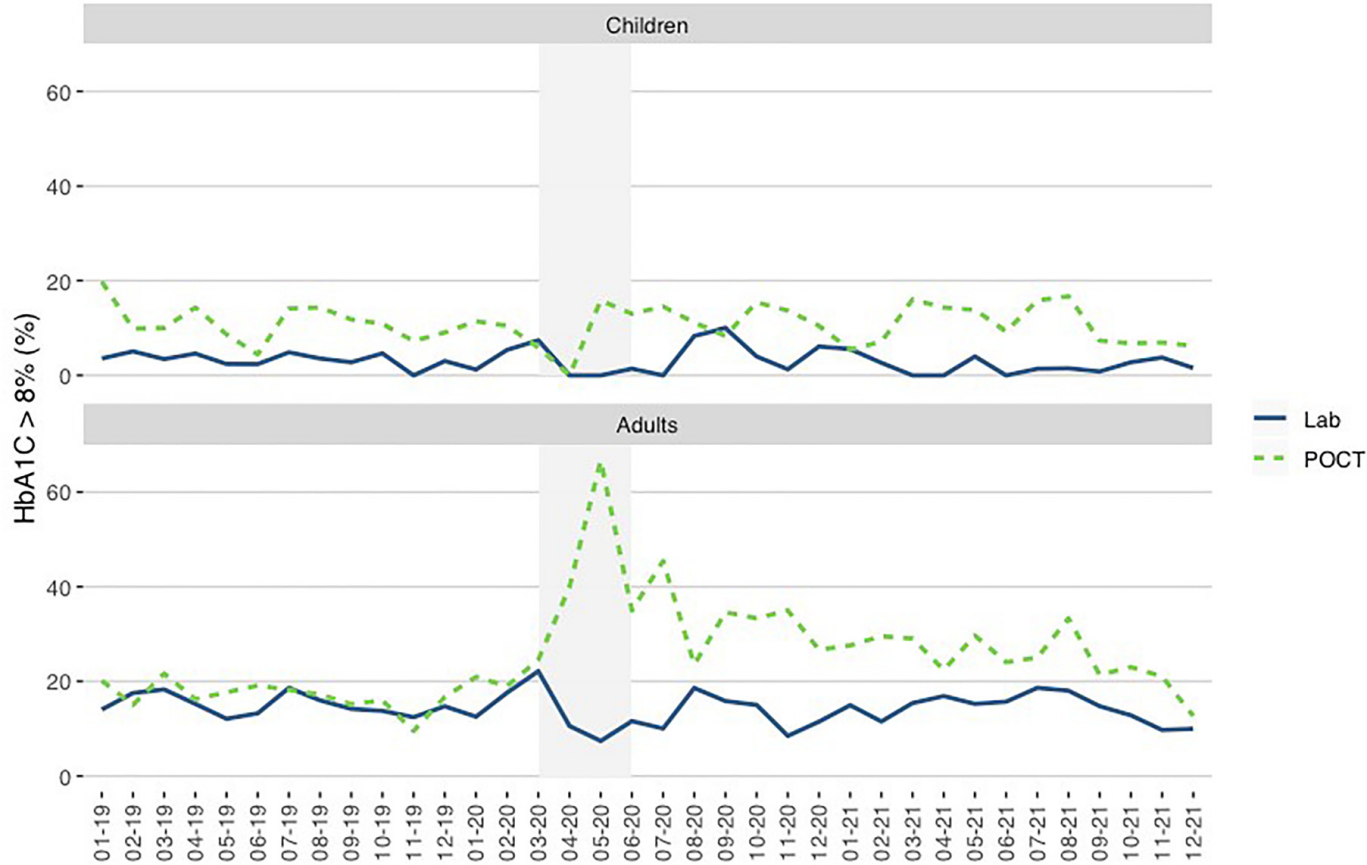
Y axis: Percentage of HbA_1c_ >8% obtained in pediatric and adult patients in the laboratory and POCT. X axis: Time (month–year). The period of strict lockdown in Madrid corresponds to the shaded area (March–May 2020).

The HbA_1c_ results above 8% (63.9 mmol/mol IFCC) obtained in pediatric and adult patients are shown in Figures 5 and 6.

In general, the percentage of HbA_1c_ results above 8% remained stable during the study period. During the strict lockdown, the results decreased in both populations.

During the strict lockdown, no pediatric patients with HbA_1c_ results above 8% were found until May 2020, when four patients with HbA_1c_ results above 8% were obtained by POCT in the pediatric diabetes doctor’s office.

In adult patients, the percentage of HbA_1c_ results above 8% remained stable in the laboratory, even during strict lockdown. However, we observed an increase in POCT of 40% in April and 67% in May 2020.

## Discussion

This study includes HbA_1c_ measurements performed both in the laboratory and in POCT to have an overview of pediatric and adult diabetes patient care. Most publications are focused on laboratory measurements, without including POCT [5]. The development of a consolidated and accredited POCT network in our hospital over 24 years has allowed us to monitor the complete testing process, detecting deviations that could have an impact on patient care.

During the lockdown (March–May 2020), the majority of those with diabetes attended telemedicine visits, without loss to follow-up. It explains the significant decrease in the total number of HbA_1c_ measurements observed during this period. The in-hospital visits started gradually again from June 2020. The return to the pre-pandemic situation was slower in adults because life-threatening and critical cases of COVID-19 have been found to be more frequent among older patients or in persons with comorbidities such as DM, hypertension, or cancer [1]. These patients preferred to attend the hospital’s blood draw ward for sample collection to be sent to the laboratory and to subsequently attend the telemedicine visit.

Regarding the HbA_1c_ results, we did not observe higher results in the post-pandemic period. Approximately 45% of the adult patients and 90% of the pediatric patients with type 1 DM had been monitored with continuous glucose since 2020. Despite the difficulties of the COVID-19 pandemic, this telemedicine monitoring and a high level of diabetes self-management helped to maintain and even improve metabolic control. Similar results have been observed in other studies [7]. Luzi et al. [8] detected a reduction of HbA_1c_ during the lockdown period in patients who used continuous glucose monitoring as a method for diabetes management during that time. Alharthi et al. [9] noticed that those who had attended a telemedicine visit during the lockdown period had a significant improvement in glycemic metrics. In contrast, there were no significant changes in those who did not attend telemedicine. These results support the clinical effectiveness of telemedicine in diabetes care.

Although no evidence has been observed of a negative association between the decrease in outpatient visits and glycemic control in 2020, some concerns remain about possible effects on screening, follow-up, and glycemic control, also for type 2 DM, given that a large number of in-person visits were postponed during the pandemic and the access to telemedicine could be different in regard to some populations, creating possible barriers to access. There is a need to recover in-person visits for all patients with chronic conditions to ensure proper follow-up and control and to prevent future negative outcomes [2].

In contrast, other studies have observed a loss of glycemic control and subsequent complications during the lockdown period [1]. Along these lines, they detected an increase in HbA_1c_ from baseline of 2.26% after 30 days of lockdown and 3.68% after 45 days of lockdown. The projected increase in the complication rate was 2.9% for peripheral neuropathy, 2.8% for diabetic retinopathy, 0.5% for stroke, 0.9% for heart attacks, and 14.2 and 9.3% for proteinuria and microalbuminuria, respectively. This change represented an increase in the number of people with DM and its complications [1]. Pardhan et al. [4] observed that self-isolation was shown to impact almost all factors that influence diabetes self-management. A targeted approach to improve access to diabetes medicine and healthy diets for people who need to self-isolate is vital to ensure that they are able to self-manage their diabetes effectively.

Focusing on pediatric patients, Predieri et al. [10] involved patients with type 1 DM using real-time continuous glucose monitoring before the schools’ closure and from the consecutive lockdown. The glycemic control improved during lockdown. Despite that patients were confined to their homes and limited in exercise, the use of real-time continuous glucose monitoring, continuous parental management and telemedicine showed beneficial effects on patient care. Similar results were obtained by Hakonen et al. [11]. The glycemic control in children with type 1 DM did not deteriorate during the lockdown, and patients on insulin pumps even improved their control, which suggests that social distancing might have allowed families to use the insulin pump more accurately, given that out-of-home activities were on hold.

Finally, the increase in HbA_1c_ results above 8% observed in our study for adults in POCT in April and May 2020 was related to patients with severe clinical situation who continued to be attended in the emergency department: new diabetes onset, ketoacidosis or hyperosmolar crisis. These patients were directly sent to the diabetes unit for an in-person visit.

## Conclusions

In summary, the management of patients with diabetes during the COVID-19 pandemic has been a great challenge. In our hospital, continuous glucose monitoring and a telemedicine approach have been crucial, even allowing for improvement of the HbA_1c_ results. During the lockdown, patients with better metabolic control were managed from the laboratory. On the other hand, patients with poorer control or a severe clinical situation underwent POCT tests in the diabetes units for measuring HbA_1c_ as POCT as needed. Adults returned to pre-pandemic in-person management more slowly because they were more susceptible to morbidity and mortality due to COVID-19 and more precautions had been adopted. Coordination between all health professionals has been a fundamental pillar to offering the best management possible, which is focused on the clinical status of patients, especially in challenging scenarios such as the COVID-19 pandemic. Adequate use of a combination of laboratory methods and POCT for HbA_1c_ monitoring together with novel management approaches such as telemedicine contributed to achieving these positive results.

## Supplementary Material

Supplementary Material

## References

[1] Verma AK, Ali Beg M, Bhatt D, Dev K, Alsahli MA, Rahmani AH (2021). Assessment and management of diabetic patients during the COVID-19 pandemic. Diabetes Metab Syndr Obes.

[2] Coma E, Miró Q, Medina M, Marin-Gomez FX, Cos X, Benitez M (2021). Association between the reduction of face-to-face appointments and the control of patients with type 2 diabetes mellitus during the Covid-19 pandemic in Catalonia. Diabetes Res Clin Pract.

[3] Bain SC, Czernichow S, Bogelund M, Madsen ME, Yssing C, McMillan AC (2021). Costs of COVID-19 pandemic associated with diabetes in Europe: a health care cost model. Curr Med Res Opin.

[4] Pardhan S, Islam MS, Lopez-Sanchez GF, Upadhyaya T, Sapkota RP (2021). Self-isolation negatively impacts self-management of diabetes during the coronavirus (COVID-19) pandemic. Diabetol Metab Syndr.

[5] Oliver P, Fernandez-Calle P, Mora R, Diaz-Garzon J, Prieto D, Manzano M (2020). Real-world use of key performance indicators for point-of-care testing network accredited by ISO 22870. Pract Lab Med.

[6] Aarsand AK, Fernandez-Calle P, Webster C, Coskun A, Gonzales-Lao E, Diaz-Garzon J (Jun). The EFLM biological variation database.

[7] Moreno-Domínguez O, González-Pérez de Villar N, Barquiel B, Hillman-Gadea N, Gaspar-Lafuente R, Arevalo-Gomez M (2021). Factors related to improvement of glycemic control among adults with type 1 diabetes during lockdown due to COVID-19. Diabetes Technol Ther.

[8] Luiz L, Carruba M, Crialesi R, Da Empoli S, Dagani R, Lovati E (2021). Telemedicine and urban diabetes during COVID-19 pandemic in Milano, Italy during lock-down: epidemiological and sociodemographic picture. Acta Diabetol.

[9] Alharthi SK, Alyusuf EY, Alguwaihes AM, Alfadda A, Al-Sofiani ME (2021). The impact of a prolonged lockdown and use of telemedicine on glycemic control in people with type 1 diabetes during the COVID-19 outbreak in Saudi Arabia. Diabetes Res Clin Pract.

[10] Predieri B, Leo F, Candia F, Lucaccioni L, Madeo SF, Pugliese M (2020). Glycemic control improvement in Italian children and adolescents with type 1 diabetes followed through telemedicine during lockdown due to the COVID-19 pandemic. Front Endocrinol.

[11] Hakonen E, Varimo T, Tuomaala A, Miettinen PJ, Pulkkinen MA (2022). The effect of COVID-19 lockdown on the glycemic control of children with type 1 diabetes. BMC Pediatr.

