# Supplementary material for: Impact of COVID-19 pandemic on HbA1c management and results in pediatric and adult outpatients with diabetes
Source: Adv Lab Med. 2023 Feb 28;4(1):105–11. doi: 10.1515/almed-2022-0098 (PMC10197181; doi:10.1515/almed-2022-0098)
Supplement: Supplementary file 1 — Supplementary Material [file j_almed-2022-0098_suppl_001.docx]

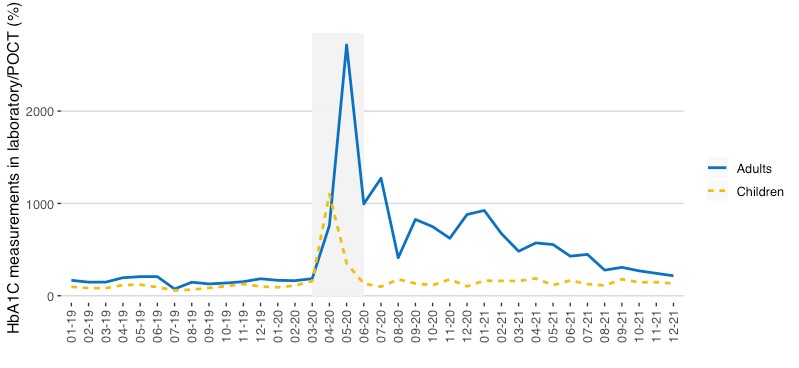


Supplemental Figure 1. Y axis: Ratio of HbA1c measurements performed in the laboratory / POCT (%) in each clinical setting. X axis: Time (month-year). The period of strict lockdown in Madrid corresponds to the shaded area (March-May 2020).
